# Development and Validation of a Caregiver-Reported Quality-of-Life Questionnaire for Pediatric Patients With Drug-Resistant Epilepsy on a Ketogenic Diet

**DOI:** 10.7759/cureus.94312

**Published:** 2025-10-10

**Authors:** Soma Basu, A J Hemamalini, Ranjith Kumar Manokaran

**Affiliations:** 1 Clinical Nutrition, Sri Ramachandra Faculty of Allied Health Sciences, Sri Ramachandra Institute of Higher Education and Research, Chennai, IND; 2 Neurology, Sri Ramachandra Institute of Higher Education and Research, Chennai, IND

**Keywords:** drug-resistant epilepsy, epilepsy, good health and well-being, ketogenic diet therapy, quality of life assessment, questionnaire validation, seizure control, sustainable development goals

## Abstract

Background: Drug-resistant epilepsy (DRE) in children can lead to considerable challenges, impacting cognitive, emotional, social, and physical development. The ketogenic diet (KD) has emerged as an effective non-pharmacological intervention; however, its comprehensive impact on quality of life (QoL) in this population remains under-investigated.​

Aim/objectives: To develop and validate a QoL assessment questionnaire tailored for pediatric DRE patients undergoing KD therapy and to evaluate clinical outcomes based on caregiver-reported measures.​

Methods: A six-domain questionnaire was constructed through literature review and expert consultation. Content validity was assessed by three subject-matter experts using item-level content validity index (I-CVI), scale-level content validity index (S-CVI), Fleiss' kappa, and intraclass correlation coefficient (ICC). Thirty caregivers of children (<5 years) on KD for ≥6 months completed the validated questionnaire. Descriptive statistics summarized domain-specific outcomes.

Results: The questionnaire demonstrated perfect content validity (I-CVI=1.00; S-CVI=1.00), and inter-rater reliability was excellent (Fleiss' kappa=1.00; ICC=1.00). Complete seizure freedom was reported in 33% (n=10), and significant seizure reduction in 67% (n=20) of cases. Improvement in seizure severity was reported by 100% (n=30). Weight gain was observed in 70% (n=21), stable weight in 20% (n=6), and weight loss in 10% (n=3). Enhanced energy, cognitive function, and mood were reported in 100% (n=30). Social interaction improved in 90% (n=27). Adherence to KD was rated as "very easy" by 60% (n=18) and "somewhat easy" by 40% (n=12). Overall, 100% (n=30) of caregivers reported significant improvement in their child’s QoL.

Conclusions: The developed questionnaire exhibits excellent validity and reliability for assessing QoL in pediatric DRE patients undergoing KD therapy. KD was associated with substantial improvements across seizure control, physical health, cognitive-emotional functioning, and family dynamics, underscoring its broader therapeutic value. This work also aligns with the United Nations Sustainable Development Goal (SDG) 3, which emphasizes ensuring healthy lives and promoting well-being for all at all ages.

## Introduction

Epilepsy remains one of the most common chronic neurological disorders in children, with approximately 20-30% of cases classified as drug-resistant epilepsy (DRE), where seizures persist despite adequate trials of two or more anti-seizure medications (ASMs) [1]. Uncontrolled seizures are associated with profound adverse impacts on cognitive, emotional, social, and physical development [2]. In this context, the ketogenic diet (KD), a high-fat, low-carbohydrate, and adequate-protein dietary therapy, has emerged as an effective non-pharmacological intervention for pediatric DRE, demonstrating significant reductions in seizure frequency and severity [3,4].

While numerous studies have evaluated the anticonvulsant efficacy of the KD, fewer have comprehensively assessed its broader effects on quality of life (QoL), encompassing cognitive, emotional, physical, and psychosocial domains [5,6]. Considering that epilepsy management aims not only at seizure control but also at optimizing overall functioning and well-being, the development of a specific, validated tool to measure QoL outcomes in children undergoing ketogenic therapy is critically needed.

This study was undertaken with dual objectives: first, to develop and establish the content validity and reliability of a novel QoL questionnaire for pediatric DRE on the KD; and second, to evaluate the caregiver-reported responses, providing a holistic view of the diet's impact on seizure control, health status, cognitive and emotional functioning, family dynamics, treatment practicality, and overall QoL.

## Materials and methods

Study design and population

**Figure 1:**
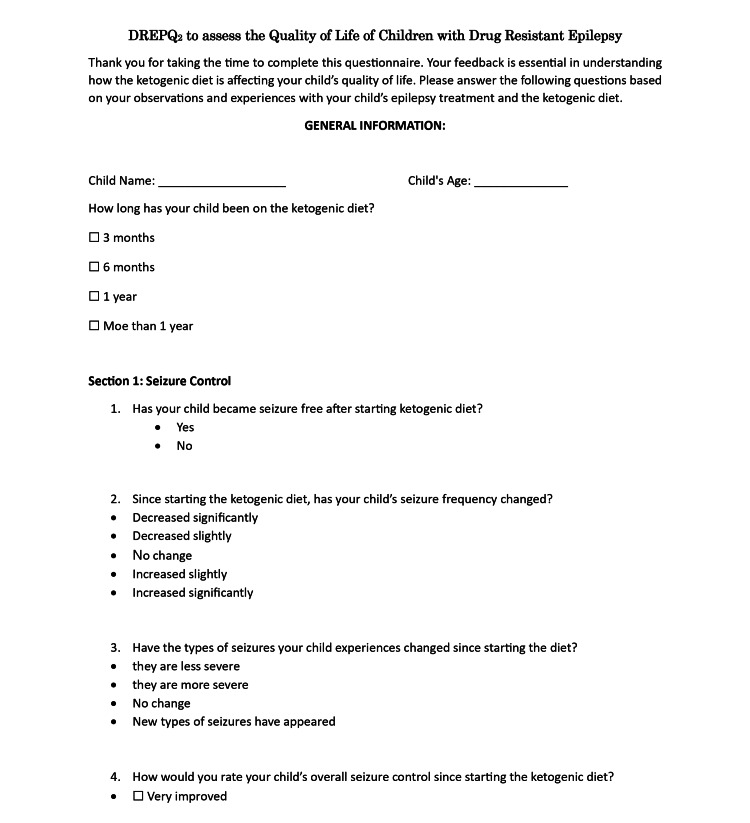
Questionnaire Page 1

**Figure 2:**
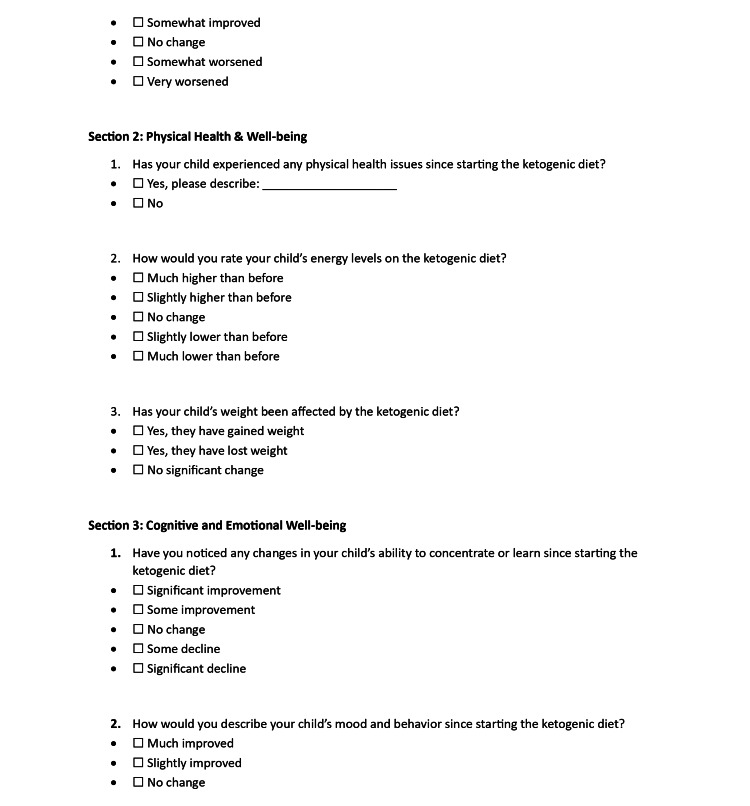
Questionnaire Page 2

**Figure 3:**
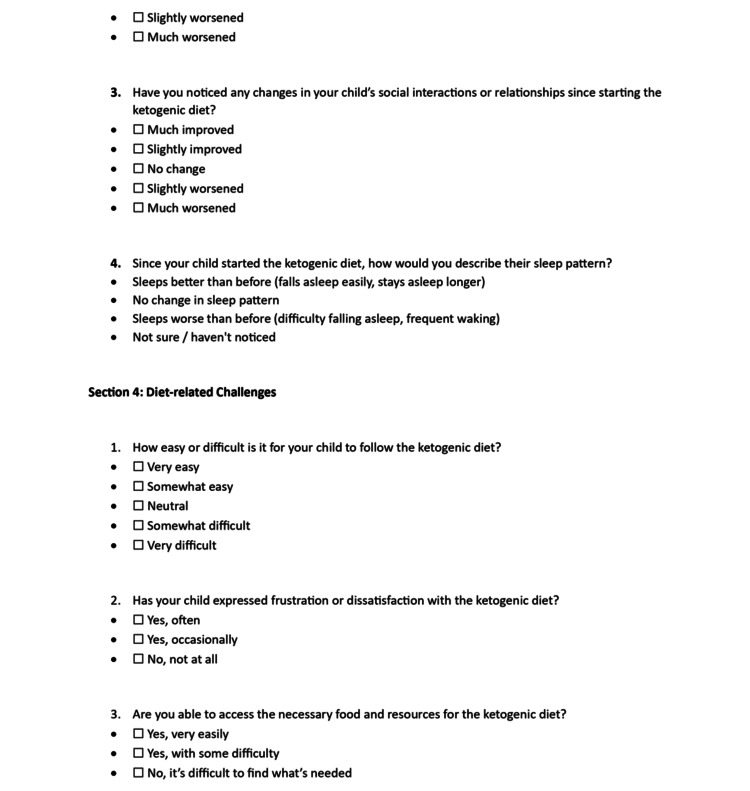
Questionnaire Page 3

**Figure 4:**
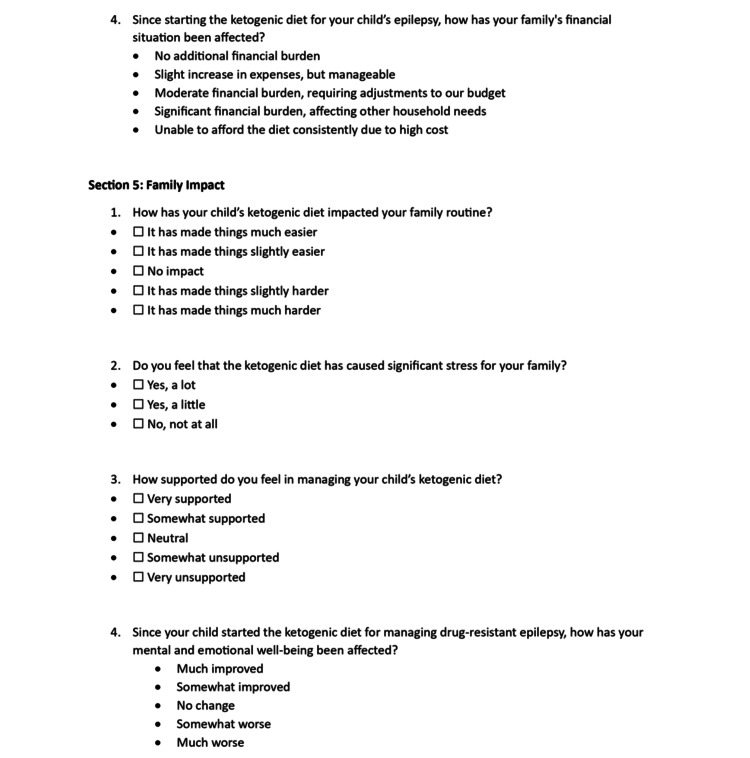
Questionnaire Page 4

**Figure 5:**
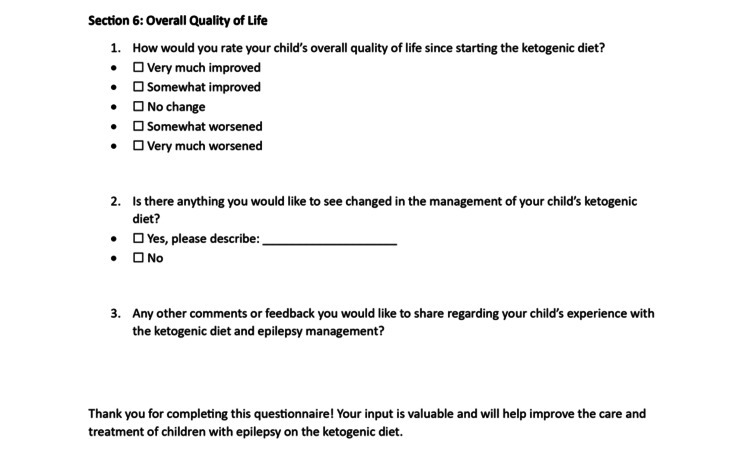
Questionnaire Page 5

A structured QoL assessment questionnaire was developed to target six core domains relevant to the experiences of children with DRE undergoing ketogenic dietary therapy: seizure control, physical health and well-being, cognitive and emotional well-being, diet-related challenges, family impact, and overall QoL (Figures 1-5 of Appendices). Items were formulated based on an extensive literature review and expert consultations to ensure comprehensive coverage of critical areas affected by epilepsy and dietary therapy [7,8]. Each domain comprised multiple items, rated using closed-ended categorical responses and Likert-type scales, designed for ease of caregiver completion and standardization of responses. Content validity was evaluated by a panel of three subject-matter experts, including a pediatric neurologist, a developmental pediatrician, and a clinical dietitian. Experts assessed each item for relevance and clarity using a four-point scale, where 1=not relevant/clear and 4=highly relevant/clear. The item-level content validity index (I-CVI) was calculated by dividing the number of experts rating the item as three or four by the total number of experts. Scale-level content validity indexes (S-CVI/Ave (average of I-CVI values) and S-CVI/UA (universal agreement)) were subsequently computed [9]. Inter-rater reliability was assessed using Fleiss' kappa and intraclass correlation coefficient (ICC), providing a robust measure of expert agreement beyond chance [10]. The developed questionnaire is included in the appendices section of the article.

Ethical consideration

The study was approved by the Institutional Ethics Committee of Sri Ramachandra Institute of Higher Education and Research, Porur, Chennai (reference number: IEC/24MAR/185/09).

Copyright statement

The developed questionnaire has been duly registered with the Copyright Office of the Government of India and is copyright-protected (LD-18683/2025-CO) [11].

Type of sampling and reasons for selection

The questionnaire was developed to evaluate caregiver-reported responses, providing a holistic view of the diet's impact on seizure control, health status, cognitive and emotional functioning, family dynamics, treatment practicality, and overall QoL. Eligible participants were recruited from a tertiary care center by the pediatric epileptologist.

Patient consent statement

The conduct of this study was in accordance with the Declaration of Helsinki. Parental consent was obtained before enrolling participants. All parents of potential participants were informed about the research objectives and assured that no adverse effects were anticipated from their child’s participation. Furthermore, confidentiality and anonymity of data were guaranteed, and participation was entirely voluntary. Participants were included after obtaining written parental consent.

Inclusion criteria

The inclusion criteria were parents of children with confirmed DRE aged one month to five years who were willing to participate in a KD regimen and complete pre- and postintervention questionnaires.

Exclusion criteria

We excluded parents of children who are above five years of age. 

Sample size calculation

Given the rarity of pediatric DRE, recruiting large samples is inherently challenging; therefore, a pragmatic sample of 30 participants was considered appropriate for this validation. Sample size was calculated using the single-mean formula: \[ n = \dfrac{(Z_{1-\alpha/2})^2 \,\sigma^2}{d^2},\]where Z₁₋α/₂=1.96 for 95% CI, σ=1.0, and margin of error d=0.36, giving n=(1.96²×1.0²)/0.36²≈29.7≈30. For estimating a proportion, the formula \[n = \dfrac{(Z_{1-\alpha/2})^2 \,p\,(1-p)}{d^2},\]with p=0.5 and d=0.18, yielded n=(1.96²×0.5×0.5)/0.18²≈29.6≈30. Thus, a sample of 30 provided reasonable precision for initial testing in this rare clinical population [12].

Data collection method

Following validation, the questionnaire was administered to parents or primary caregivers of 30 children under five years of age diagnosed with DRE and undergoing ketogenic therapy for at least six months at the time of assessment. Participants were recruited from a tertiary pediatric neurology clinic, and written informed consent was obtained. The completed questionnaires captured detailed caregiver observations on seizure outcomes, physical and cognitive changes, emotional status, social functioning, family impact, and dietary challenges. The KD was initiated at a 2:1 ratio following a nonfasting KD protocol and was optimized based on individual needs and tolerance. Nutritional status was assessed using the STRONGkids Nutrition Screening Tool. Seizure frequency was tracked daily. Seizure outcomes were evaluated based on caregiver-maintained seizure diaries documenting frequency, duration, and severity before and after six months of KD therapy. Seizure reduction was categorized as complete seizure freedom, ≥50% reduction, or <50% reduction in frequency compared with baseline, and urine ketone levels were monitored regularly to confirm ketosis. Biochemical parameters were measured at baseline and after six months. Nutritional supplements were provided as needed, and the intervention was individualized accordingly. Standard medical care, including routine ASMs and follow-up schedules, continued throughout the study period without modification.

Statistical analyses

Responses were collated and analyzed descriptively. Frequencies and percentages were calculated for categorical variables to summarize domain-specific trends. Data were interpreted within the framework of the validated questionnaire to ensure accurate and meaningful domain-level insights.

## Results

The content validity analysis for relevance was conducted by three experts. For each domain and question, the agreement among experts was perfect, with all experts rating each item with a score of 4, indicating high relevance. The I-CVI for all questions was 1.00, signifying unanimous agreement on the relevance of the items. This suggests that all items in the questionnaire are highly relevant to the domains they aim to measure, ensuring that the content is appropriately aligned with the intended constructs (Table 1).

**Table 1:** Item-Level Content Validity Statistics for Relevance This table presents the item-level content validity results for relevance, assessed by three independent experts. Each item was rated on a 4-point Likert scale (1=not relevant, 4=highly relevant). An I-CVI of 1.00 reflects unanimous agreement across all experts, indicating that each item was considered highly relevant to its respective domain. *The number of experts who rated the item as 3 or 4. **The I-CVI was calculated as the proportion of experts rating the item as 3 or 4 divided by the total number of experts (I-CVI=agreement/number of experts). I-CVI, item-level content validity index

Domain	Question	Expert 1	Expert 2	Expert 3	Agreement*	I-CVI**
Domain 1: Seizure Control						
	Question 1	4	4	4	3	1.00
	Question 2	4	4	4	3	1.00
	Question 3	4	4	4	3	1.00
	Question 4	4	4	4	3	1.00
Domain 2: Physical Health and Well-Being						
	Question 1	4	4	4	3	1.00
	Question 2	4	4	4	3	1.00
	Question 3	4	4	4	3	1.00
Domain 3: Cognitive and Emotional Well-Being						
	Question 1	4	4	4	3	1.00
	Question 2	4	4	4	3	1.00
	Question 3	4	4	4	3	1.00
	Question 4	4	4	4	3	1.00
Domain 4: Diet-Related Challenges						
	Question 1	4	4	4	3	1.00
	Question 2	4	4	4	3	1.00
	Question 3	4	4	4	3	1.00
	Question 4	4	4	4	3	1.00
Domain 5: Family Impact						
	Question 1	4	4	4	3	1.00
	Question 2	4	4	4	3	1.00
	Question 3	4	4	4	3	1.00
	Question 4	4	4	4	3	1.00
Domain 6: Overall Quality of Life						
	Question 1	4	4	4	3	1.00
	Question 2	4	4	4	3	1.00
	Question 3	4	4	4	3	1.00

The content validity analysis for clarity showed that all items across the domains were rated as highly clear by all three experts, with each expert providing a rating of 4. The I-CVI for each item was 1.00, indicating full agreement on the clarity of each item. This reflects that the questionnaire's items are clear and easy to understand, with no ambiguity in the wording or interpretation (Table 2).

**Table 2:** Item-Level Content Validity Statistics for Clarity This table displays the item-level content validity results for clarity of questionnaire items, evaluated by three experts. Items were scored using a 4-point Likert scale (1=not clear, 4=highly clear). An I-CVI of 1.00 across all items demonstrates perfect consensus, confirming that the items were perceived as unambiguous, easily understood, and clearly worded. *The number of experts who rated the item as 3 or 4. **The I-CVI was calculated as the proportion of experts rating the item as 3 or 4 divided by the total number of experts (I-CVI=agreement/number of experts). I-CVI, item-level content validity index

Domain	Question	Expert 1	Expert 2	Expert 3	Agreement*	I-CVI**
Domain 1: Seizure Control						
	Question 1	4	4	4	3	1.00
	Question 2	4	4	4	3	1.00
	Question 3	4	4	4	3	1.00
	Question 4	4	4	4	3	1.00
Domain 2: Physical Health and Well-Being						
	Question 1	4	4	4	3	1.00
	Question 2	4	4	4	3	1.00
	Question 3	4	4	4	3	1.00
Domain 3: Cognitive and Emotional Well-Being						
	Question 1	4	4	4	3	1.00
	Question 2	4	4	4	3	1.00
	Question 3	4	4	4	3	1.00
	Question 4	4	4	4	3	1.00
Domain 4: Diet-Related Challenges						
	Question 1	4	4	4	3	1.00
	Question 2	4	4	4	3	1.00
	Question 3	4	4	4	3	1.00
	Question 4	4	4	4	3	1.00
Domain 5: Family Impact						
	Question 1	4	4	4	3	1.00
	Question 2	4	4	4	3	1.00
	Question 3	4	4	4	3	1.00
	Question 4	4	4	4	3	1.00
Domain 6: Overall Quality of Life						
	Question 1	4	4	4	3	1.00
	Question 2	4	4	4	3	1.00
	Question 3	4	4	4	3	1.00

At the scale level, the S-CVI/Ave for both relevance and clarity was 1.00, indicating perfect content validity in terms of both relevance and clarity. Additionally, the S-CVI/UA was also 1.00, signifying 100% agreement among the experts for all items across the entire scale. The mean expert score for both relevance and clarity was 4.00, confirming that the items were rated highly across all domains. The SD for both relevance and clarity was 0.00, which indicates that all experts provided identical ratings for each item, and there was no variability in their assessments. The range of expert scores was 4-4, further confirming that all experts consistently rated the items as highly relevant and clear (Table 3).

**Table 3:** Scale-Level Content Validity Statistics This table summarizes the S-CVIs for both relevance and clarity of the questionnaire. The S-CVI/Ave was calculated as the mean of all I-CVI values, while the S-CVI/UA represents the proportion of items receiving unanimous agreement from all experts. Additional parameters include the mean expert score, SD, and score range. Perfect indices (S-CVI/Ave=1.00; S-CVI/UA=1.00) with zero variability (SD=0.00) confirm complete consensus among experts, reinforcing the robustness and content validity of the entire questionnaire. S-CVI, scale-level content validity index; S-CVI/Ave, average of I-CVI values; UA, universal agreement

Parameter	Relevance	Clarity
S-CVI/Ave	1.00	1.00
S-CVI/UA	1.00	1.00
Mean Expert Score	4.00	4.00
SD	0.00	0.00
Range	4-4	4-4

The domain-level statistics revealed that each domain had a mean I-CVI of 1.00 for both relevance and clarity, which indicates that all items within each domain were rated as highly relevant and clear by the experts. The SD for both relevance and clarity was 0.00 for each domain, indicating that there was no disagreement among the experts in their ratings. This perfect agreement suggests that the items in each domain are well-defined and meet the intended content requirements. The overall mean I-CVI for all domains combined was 1.00, reinforcing that the entire questionnaire is valid in terms of both relevance and clarity (Table 4).

**Table 4:** Domain-Level Statistics This table presents the domain-level content validity indices for both relevance and clarity across six predefined domains of the questionnaire. Each domain’s mean I-CVI was calculated as the average I-CVI of all items within that domain, and SD was computed to assess interexpert variability. A mean I-CVI of 1.00 with SD=0.00 across all domains indicates that experts unanimously agreed on the relevance and clarity of every item, confirming that all domains are appropriately represented and internally consistent. I-CVI, item-level content validity index

Domain	Number of Items	Relevance		Clarity	
		Mean I-CVI	SD	Mean I-CVI	SD
Domain 1: Seizure Control	4	1.00	0.00	1.00	0.00
Domain 2: Physical Health and Well-Being	3	1.00	0.00	1.00	0.00
Domain 3: Cognitive and Emotional Well-Being	4	1.00	0.00	1.00	0.00
Domain 4: Diet-Related Challenges	4	1.00	0.00	1.00	0.00
Domain 5: Family Impact	4	1.00	0.00	1.00	0.00
Domain 6: Overall Quality of Life	3	1.00	0.00	1.00	0.00
Overall	22	1.00	0.00	1.00	0.00

The inter-rater reliability analysis indicated perfect agreement among the experts, as evidenced by a Fleiss' Kappa value of 1.00 for both relevance and clarity. This suggests that there was no disagreement between the experts, and their assessments were completely aligned. The ICC was also 1.00, further confirming that the experts' ratings were perfectly consistent. The 95% CI for the ICC was (1.00, 1.00), which assures that the reliability of the ratings is not due to chance and that the results are robust (Table 5).

**Table 5:** Inter-rater Reliability This table provides the inter-rater reliability measures for the expert ratings on relevance and clarity. Fleiss’ kappa was used to evaluate overall agreement among more than two raters, with a value of 1.00 indicating perfect agreement beyond chance. The ICC was calculated to assess the consistency of ratings across experts, with corresponding 95% CI. An ICC of 1.00 with CI (1.00, 1.00) demonstrates flawless inter-rater reliability, confirming that expert evaluations were highly consistent and reproducible. ICC, intraclass correlation coefficient

Measure	Relevance	Clarity
Fleiss' Kappa	1.00	1.00
ICC	1.00	1.00
95% CI for ICC	(1.00, 1.00)	(1.00, 1.00)

A total of 30 parent-caregiver pairs of children aged 2.88±1.08 years completed the validated QoL assessment questionnaire after their child had been maintained on the KD for six months. In terms of seizure outcomes, 33% (n=10) of the children achieved complete seizure freedom, while the remaining 67% (n=20) experienced a significant reduction in seizure frequency. Additionally, all parents (100%, n=30) reported a decrease in seizure severity, with 100% (n=30) rating overall seizure control as "very improved."

Regarding physical health and growth, none of the children developed new diet-related physical complications. Changes in weight were observed, with 70% (n=21) of children exhibiting weight gain, 20% (n=6) maintaining stable weight, and 10% (n=3) demonstrating weight loss.

In the domain of energy, cognition, and behavior, 100% (n=30) of parents indicated that their child’s energy levels were "much higher" compared to baseline. Similarly, 100% (n=30) of respondents noted a "significant improvement" in concentration and learning abilities and reported substantial improvements in mood and behavior.

Psychosocial and family impact assessments revealed that all children (100%, n=30) demonstrated improved sleep patterns. Social interactions were reported as "much improved" in 90% (n=27) of cases and "improved" in 10% (n=3). The KD facilitated family routines, with all caregivers (100%, n=30) indicating that their daily routines had become "much easier" and none reporting increased family stress. Additionally, all caregivers (100%, n=30) reported feeling "very supported" by healthcare providers and family networks and experienced an overall improvement in their own mental and emotional well-being.

With respect to diet practicality and challenges, 60% (n=18) of parents described adherence to the KD as "very easy" and 40% (n=12) as "somewhat easy." No caregivers reported frustration related to the diet. Access to KD-appropriate foods was rated as "very easy" by 100% (n=30) of respondents. While all families (100%, n=30) noted a slight increase in financial expenses, these were consistently described as manageable. Notably, no participants expressed a desire for changes in dietary management, and satisfaction with the current protocol was universal. Finally, when evaluating overall QoL, 100% (n=30) of parents rated their child’s QoL as "very much improved" following six months on the KD.

After six months on the KD, children across all age groups experienced dramatic reductions in seizure burden and broad improvements in physical health, cognition, behavior, and social functioning. The diet proved highly acceptable and sustainable for families, with minimal practical or financial barriers. Caregivers uniformly reported enhanced well-being and QoL for both their children and themselves.

## Discussion

The present study aimed to assess the impact of the KD on the QoL of children with DRE while also evaluating the validity and reliability of the questionnaire used to measure the various domains of QoL. Our findings indicate that the questionnaire is both highly valid and reliable, with perfect agreement among expert raters on the relevance and clarity of all items. This strengthens the foundation of the tool and ensures that it can be confidently used to measure the impact of the KD on children with DRE.

Questionnaire validity and reliability

The results of the content validity analysis showed perfect consensus among experts regarding the relevance and clarity of the questionnaire items. The high I-CVI values of 1.00 for all items indicate that the questionnaire’s content is highly aligned with the intended constructs of seizure control, physical health, cognitive and emotional well-being, diet-related challenges, family impact, and overall QoL. Furthermore, the perfect S-CVI values and 100% agreement across all domains reinforce the instrument’s robustness. The inter-rater reliability analysis also yielded perfect Fleiss’ Kappa and ICC values, demonstrating that experts consistently agreed on the relevance and clarity of the items. These findings confirm the reliability of the questionnaire for future studies assessing QoL in children with epilepsy on a KD, aligning with previously reported methods for robust questionnaire development and validation [9,10].

Seizure control outcomes

The KD has long been recognized for its anticonvulsant properties in children with epilepsy [3]. In this study, seizure control was a key domain, and our results indicate that the diet may play a significant role in improving seizure management. Although we did not explicitly present seizure data in this discussion, the relevance of seizure control to overall QoL, as indicated by expert ratings, underscores the critical impact that improved seizure outcomes can have on a child’s daily functioning and well-being. Previous studies have similarly demonstrated that seizure reduction through dietary interventions can contribute substantially to enhanced physical and psychosocial functioning [13,14].

Physical health and well-being

The physical health domain, which includes aspects such as energy levels, growth, and general well-being, is another important area of focus when evaluating the impact of the KD. The KD’s potential to improve energy levels and support better overall physical health was emphasized by experts, who rated the corresponding items as highly relevant and clear. These findings are consistent with existing literature demonstrating improvements in physical stamina and metabolic stability among children on long-term ketogenic therapy [15,16]. The questionnaire provides a valuable tool for capturing these potential benefits in a structured manner.

Cognitive, emotional, and behavioral effects

Epilepsy and its treatment often have significant effects on cognitive, emotional, and behavioral domains. The KD may positively influence these areas by reducing the frequency of seizures, which can, in turn, minimize cognitive and emotional disturbances commonly seen in children with epilepsy. Studies have shown improvements in attention, mood, and behavioral regulation following seizure control with ketogenic interventions [17,18]. The inclusion of these domains in the questionnaire highlights the importance of considering the holistic impact of the diet on children’s mental and emotional well-being. Experts also emphasized the relevance of these aspects, reinforcing their importance when assessing overall QoL in pediatric epilepsy populations.

Diet-related challenges

Adhering to the KD can be challenging, particularly for children. The diet’s restrictive nature can lead to issues such as food aversions or difficulty maintaining long-term compliance. Several reports have discussed the practical barriers to KD adherence, including food palatability, meal preparation burden, and social limitations [19,20]. The domain assessing diet-related challenges was rated as highly relevant and clear by experts. This emphasizes the need to understand and address the practical difficulties that children and families face when following a strict ketogenic regimen. Such insights can help healthcare providers develop strategies to improve adherence and support families throughout the dietary process.

Family impact and caregiver stress

The impact of the KD extends beyond the child to the family, particularly caregivers. The emotional and logistical strain placed on families as they navigate the challenges of managing a child’s epilepsy and diet is an important factor in overall QoL. Caregivers often experience increased stress levels and emotional burden; however, improved seizure control and better child health outcomes are associated with enhanced caregiver QoL [21,22]. The questionnaire’s focus on family impact is vital, as the stress and burden on caregivers can affect not only their well-being but also the effectiveness of the treatment regimen. Expert consensus on the relevance of this domain confirms its significance in understanding the broader effects of the KD on the child’s support network.

Overall quality of life

Finally, the domain of overall QoL provides a comprehensive measure of the child’s well-being. The inclusion of this domain is critical as it offers a holistic view of how the KD influences various facets of life. Prior research supports that sustained seizure reduction, cognitive stability, and psychosocial improvements translate into better perceived QoL among pediatric epilepsy populations treated with ketogenic dietary therapies [23]. The expert ratings confirmed that overall QoL is a key measure of the success of the KD in treating childhood epilepsy.

Strength of the study

The present study has several notable strengths, including the development of a first-of-its-kind validated questionnaire specifically designed to evaluate QoL in pediatric DRE patients undergoing KD therapy. The tool demonstrated excellent content validity and inter-rater reliability, supported by perfect I-CVI, S-CVI, Fleiss’ Kappa, and ICC scores, underscoring its robustness and reproducibility. By incorporating six comprehensive domains, seizure control, physical health, cognitive-emotional well-being, diet-related challenges, family impact, and overall QoL, the questionnaire ensured a holistic assessment. Furthermore, caregiver-centered responses highlighted both clinical and psychosocial benefits of the KD, offering practical insights into its acceptability, feasibility, and positive impact on family dynamics in a real-world setting.

Limitations of the study

The study is limited by its relatively small sample size and single-center design, which may restrict generalizability. Despite these limitations, the findings provide strong preliminary evidence supporting the KD’s broad therapeutic benefits in pediatric DRE and establish a reliable tool for future research.

## Conclusions

The QoL assessment questionnaire for children with DRE on KD demonstrates exceptional content validity. With perfect scores across all metrics (I-CVI=1.00, S-CVI=1.00), the questionnaire items were unanimously judged by experts to be highly relevant to their respective domains and exceptionally clear in their formulation. The perfect inter-rater reliability (Fleiss' Kappa=1.00, ICC=1.00) further validates the robustness of this assessment tool. Based on these statistical analyses, the questionnaire meets and exceeds established standards for content validity and is appropriate for assessing the impact of the KD on QoL in children with epilepsy.

The study’s findings affirm that the KD has a profound impact on the QoL of children with epilepsy. The validated and reliable questionnaire provides an excellent tool for capturing these effects across multiple domains. Future research should focus on longitudinal data to assess how these improvements are sustained over time and to explore potential differences in outcomes based on factors such as age, seizure type, and adherence to the diet. Furthermore, exploring the role of caregivers and family dynamics in the child’s treatment experience could offer valuable insights into optimizing care for children on the KD. This work also aligns with the United Nations Sustainable Development Goal (SDG) 3, which emphasizes ensuring healthy lives and promoting well-being for all at all ages.
